# The medical consultation through the lenses of language and social interaction theory

**DOI:** 10.1007/s10459-018-09873-2

**Published:** 2019-02-04

**Authors:** Antoon Cox, Shuangyu Li

**Affiliations:** 1 Research Group Interpreting Studies, Faculty of Arts, KU Leuven, Louvain, Belgium; 2 Brussels Institute of Applied Linguistics and Linguistics and Literary Studies, Faculty of Arts and Philosophy, Vrije Universiteit Brussel, Brussels, Belgium; 3 Centre for Medical Education, Faculty of Life Sciences and Medicine, King’s College London, London, UK

**Keywords:** Medical communication, Language and social interaction, Linguistic diversity, Clinical skills, Immigration, Diversity

## Abstract

The well-structured medical communication models that are typically described in textbooks are relevant to practice, but the actual messy interactional realities of consultations are often a far cry away from them. As a result, medical trainees frequently encounter difficulties when applying communication skills acquired during training to medical practice. This paper reflects on how clinical communication research and courses can incorporate the growing need for context-bound communication skills training. This paper illustrates how concepts from the research field of language and social interaction can facilitate the description and analysis of communication in clinical encounters, drawing on a real-life example from an increasingly common clinical scenario: a consultation in the emergency department involving a patient who does not speak the same language as the clinician. The proposed way of looking at clinical communication can enrich clinical skills training as it provides a tool to study, analyze, visualize and discuss communication from a different perspective that simultaneously accounts for interactional and clinical reasoning aspects of medical consultations.

## Motivation

The communicative challenges encountered in medical interactions are highly context-specific (Keifenheim et al. 2015, p. 2). There is a strong interdependence between content (*what* is being communicated) and process (*how* it is communicated). In the literature, the view that the medical encounter is a unique and inherently creative event, characterized by uncertainty and situatedness and requiring clinicians to continuously make adjustments and depart from prescribed patterns, is gradually gaining ground (Durning et al. 2010; Richard and Lussier 2014; Salmon and Young 2011; Zoppi and McKegney 2002). Veldhuijzen et al. (2013) discuss how, when selecting their communicative actions, clinicians consider both characteristics of individual patients and medical goals of a consultation. Haidet (2007) goes as far as saying that doctors should incorporate knowledge drawn from communication skills training and guidelines in their own personal styles in the way jazz musicians do.^1^ At the same time, research on the interaction between content and process skills remains relatively scarce (Cary and Kurtz 2013; Kurtz et al. 2003; Rosenbaum 2017).

Clinical communication training also often remains centered on content skills and the transfer of medical and diagnostic information, rather than on process skills. Clinical work and communication are challenged by the predominance of a positivist paradigm in medicine, that often foregrounds the application of previously learned biomedical knowledge (Manidis and Scheeres 2012). Crosskerry (2003) and Croskerry et al. (2017) argue that decision-making theorists in medicine have clung to normative, often robotic, models of clinical decision-making that have little practical application in the real world of decision-making. While the well-structured medical communication models that are typically described in textbooks are relevant to practice, the actual messy interactional realities of consultations are often a far cry away from them (Atkins et al. 2016; Peräkylä and Vehvilfinen 2003; Sarangi 2010, p. 175). As a result, medical trainees frequently encounter difficulties in applying the communication skills acquired during training to medical practice (Brown 2010; Van Nuland et al. 2010).

Several authors have highlighted that the often generic nature of communication skills training is problematic, in that all consultations are treated as if they required the same communication style. In reality, style needs to be adjusted to the specific circumstances and goals of the consultation, including the type of complaint or patient, and this should be a subject matter of communication training courses (Essers et al. 2011; Salmon and Young 2011; Veldhuijzen et al. 2013). To embrace the complexity of the medical encounter, Richard and Lussier (2014) and Li et al. (2016, 2017) promote the use of dialogic approaches for medical communication training. Rollnick et al. (2002) argue that in order to convince practitioners and trainees that are skeptical of communication training, communication skills should be presented as a tool to solve problems they encounter in everyday clinical practice. They plead for a context-bound approach that starts out from training participants’ daily experiences rather than from their potential deficiencies in communication.

In a progressively globalizing world, challenges of communication with an increasingly diverse patient population are burgeoning rapidly. Patients come from an ever more varied range of cultural, linguistic, and socio-economic backgrounds, with widely diverging frames of reference. This increases the risk of miscommunication (Cox 2017) and makes the need for context-bound communication skills training for medical practitioners particularly acute.

A salient question is how medical training programs can be organized in practice as to incorporate this growing need for context-bound communication skills training. This paper aims to contribute new insights on this matter. It reflects on how the field of language and social interaction (LSI) can provide a context-bound two-way bridge between the positivist paradigm in medicine, and the constructivist or dialogic nature of the consultation itself (Rollnick et al. 2002).

The paper starts out by briefly introducing the research area of LSI. Then, it illustrates how LSI can facilitate the description and analysis of communication in clinical encounters, drawing on a real-life example from a frequently encountered clinical scenario: a consultation in the emergency department involving a patient who does not speak the same language as the clinician. The illustration is followed by a brief discussion of the relevance of the presented analysis and its implications for training of students in medicine and practitioners.

## Methods: language and social interaction theory

Language and social interaction (LSI) research brings together different research disciplines that consider that language is an integral part of a social activity, rather than only a medium of communication. Seminal work by Wittgenstein (1953) in this area posited that the meaning of words should be inferred from their context and the activities during which they are produced. Wittgenstein coined the term “language games” to acknowledge that events occurring within the context of a game usually have quite different meanings from similar events occurring outside of that game. This is an important insight which implies that, to understand what is meant, it is more insightful to look at how a word is used in a particular activity than to use an explanatory definition or a generalization (Biletzki and Matar 2014; Wittgenstein 1953).

Within the LSI strand of literature, some studies have focused on medical settings. A starting point was the study by Byrne and Long (1976), who analyzed 2500 audio-recorded general practitioner consultations in New Zealand and the UK in search of recurrent patterns in medical consultations. Based on this analysis, they described six main stages in the consultation (such as the opening, the problem presentation and so on). Later research inspired by this study showed that the way the doctor organizes these stages has an impact on the patient’s acceptance of treatment recommendations; and this was a crucial insight for improving training of healthcare professionals (Mondada 2013).

Later studies by Greenfield et al. (1985) and Roter (1977) tested patient education interventions to strengthen patient involvement and participation in medical consultations, describing the ensuing processes of communication and their outcomes. Greenfield et al. (1985) found that patient education increased patient involvement, and reduced the impact of disease on patients’ functional ability 6 to 8 weeks after the medical appointment. Roter also found a positive impact on patient participation, but a negative one on patient satisfaction with the received care. He hypothesized that the negative feelings elicited by stronger patient involvement could imply that neither the patient, nor the doctor were prepared or comfortable with an active patient role at the time of the survey; and reflect the impact of increased patient awareness of information gaps.

Heritage and Maynard (2006), two sociologists specialized in the study of medical interactions, have been promoting the use of conversation analysis to analyze doctor-patient interactions since the 1980s, to study issues such as the varying dynamics of mutual understanding and the power dynamics between patients and doctors. They described a medical consultation as an interactive process organized in turns, in which the doctor and the patient alternately take the floor and as such jointly construct the interaction. Other studies have accounted more explicitly for the context outside of the direct interaction. Work in this area (e.g. by Tannen and Wallat 1987) often builds on groundbreaking research by Goffman (1959), who particularly highlighted the connection between social interaction and theatre play (see further).

This brief overview is non-exhaustive, giving just a gist of what social interaction research has looked at in relation to medical consultations. In what follows, theories from language and social interaction (LSI) are applied to a real-life example of a medical consultation, to illustrate their usefulness to understand communication processes and how to prevent or overcome miscommunication, and their ensuing relevance for medical communication training. This illustration is by no means meant to provide a normative judgement on what is good or bad practice. It is rather intended to show how an interaction can be described in richer detail and provide relevant insights for clinical communication practice and training.

## Analysis

### Presentation of the illustrative case

The considered illustration is taken from a richer set of data collected by the author in a bilingual (French/Dutch) inner city public hospital emergency department (ED) in Brussels, Belgium. The fieldwork took place in the context of research on the origin of misunderstandings in language discordant multiparty ED consultations (Cox 2017). The project focused on consultations in which the patient and the doctor did not share a common language, and where the patient was accompanied by a relative or a friend who intervened in the consultation, mostly to provide mental and/or linguistic support. The consultations were audio-recorded and relevant contextual data were collected through ethnographic methods.

The interactional characteristics of a medical consultation are complex and evolve throughout the consultation (Durning et al. 2010). Different contextual factors that relate to the patient (such as condition, familiarity with the health system, means of communication), the clinician (including the doctor’s experience and knowledge, his prior knowledge about the patient), the clinical setting (e.g. timing, the type of consultation) and the interaction between these different factors drive and shape the consultation and its communicative characteristics in an unpredictable way (Essers et al. 2011, p. 6). In what follows, each of these types of contextual factors are briefly discussed in view of their relevance for the communication process.

#### Patient-related factors

The patient (PAT) is a Pakistani man in his late twenties. As he appears not to speak Dutch, French or English, his companion does the translation and part of the talking for him. The patient’s companion (COM) is a man of about 40 years’ old. He speaks English, Urdu, and Punjabi. It is not clear how he is related to the patient. The patient sits in an inclined position on the examination table with his hand on the left-hand side of the lower back region. His companion stands next to him. The patient’s physical position, and his first indications about his health complaints provide signals about the location of his pain and a first candidate hypothesis (renal colic, triggered by a kidney stone).

#### Doctor-related factors

The ED consultant (DOC) is a male doctor in his thirties specializing in emergency medicine. He is a native Dutch speaker but talks in English in this recording, as this is the only common language between him and the patient’s companion. He has been working since 8 am that same day and is seeing this patient at the end of his shift at around 5 pm. The doctor meets the patient for the first time in the examination booth. He has no prior medical information and is not familiar with his interlocutors’ background and language skills.

#### Consultation-related factors

The medical interaction takes place during a consultation in the ED. Emergency medicine is a unique subculture within medicine (Person et al. 2012, p. 1) and characterized by uncertainty, open-endedness and multiplicity (Chisholm et al. 2001; Eisenberg et al. 2005; Engel et al. 2010; Knopp et al. 1996; Slade et al. 2015). Doctors have no prior information on which patients they will see, what their social or medical background is, and what languages they know. They cannot control their workload, as they do not know upfront how many patients will come, and when they will come. Clinicians are typically seeing and monitoring several patients at a time, and ED consultations are often distracted by internal phone calls (Chisholm et al. 2001, p. 148).

Another key feature is time pressure. As ED services are typically characterized by overcrowding, and long and tiring clinician work shifts, various components of the usual patient-doctor communication process (establishing rapport with the patient, gathering and giving information, providing comfort and collaboration) are often performed simultaneously (Knopp et al. 1996). The venue of communication is often noisy and lacking privacy (Knopp et al. 1996). Patients tend to be stressed and in pain, and find themselves in a special psychological state. Their genuine pain experience may be exacerbated by a hyper-focus on their symptoms (Lachance 2016). More than in other types of care, patients in the ED are likely to feel very insecure and anxious (Wagley and Newton 2010) and expect physicians to be empathic. Due to the complex working conditions, some have argued that the achievement of proper doctor-patient relationships in the ED is elusive, and that ED clinicians should prioritize the medical aspects of patient encounters, with less attention to relationship building. This may lead to a discrepancy at the level of expectations between doctors and patients (Lachance 2016; Lin et al. 2008).

Research has shown that medical errors in the ED often result from poor communication (Eisenberg et al. 2005). These problems can be exacerbated by communication challenges arising from language barriers. It should therefore be no surprise that the latter have been identified as a major obstacle to proper history-taking in the ED (Burley 2011).

### Analysis of the communicative dynamics using LSI

The doctor first takes the patient’s medical history and performs a clinical exam. The patient companion mediates all communication. Communication is challenged by a multidimensional language barrier: a full language barrier between the doctor and the patient and a partial language barrier between the doctor and the companion. The fact that the companion mixes Urdu and Punjabi when talking to the patient points at a potential additional (partial) language barrier between them. The companion often speaks for the patient, rather than passing on the doctor’s questions to the patient. At times the doctor accepts this, at other times he tries to renegotiate the role taken up by the patient companion. Excerpt 1 illustrates how this role negotiation unfolds.

### Excerpt 1: History taking from a patient with a kidney stone

**Table:** 

1.	DOC	Did it came ((snaps fingers)) suddenly? Or did it came little by little
2.	COM	Little by little
3.	DOC	Ask him…
4.	COM	Yes eeehhhhe, he live with me, I know…
5.	COM	He live with me
6.	DOC	Yes yes yes… Ok… But he can have other feelings than you think… So you must translate
7.	COM	Hmhm

While the verbal exchange of information is severely challenged by the presence of a multidimensional language barrier, the doctor succeeds in achieving a fair degree of certainty on the candidate diagnosis. He is helped by the strong semantic value of the patient’s non-verbal behavior as his inclined bodily position points at the possible presence of a kidney stone.

Excerpt 2 presents the doctor’s return after having made some calls to organize an operation. The patient and his companion are waiting for the doctor on a bench in the ED hall. When the doctor tries to explain to the patient that he will need to undergo an operation the next day to remove his kidney stone, he encounters resistance. Some of this resistance can be thought of as caused by miscommunication beyond the existence of a language barrier. The excerpt illustrates how the negotiation unwinds.

### Excerpt 2: Treatment negotiation with a patient who has a kidney stone

**Table:** 

1.	DOC	We will do the operation tomorrow in the morning. So, he stays in the hospital. So we can do operation tomorrow morning. Ok?
2.	COM	Tomorrow morning?
3.	DOC	Yes
4.	COM	Euh what time?
5.	DOC	I don’t know, in the morning
6.	COM	In the morning
7.	DOC	Everything fine?
8.	COM	[in Urdu] Euh. Is it ok?
9.	COM	[in Pakistani Punjabi] So, what should we say to him?
10.	COM	[in Urdu] Qamar has to stay here
11.	PAT	[says something incomprehensible in Urdu]
12.	COM	He said euuh “tomorrow I come home?”
13.	DOC	No no no, he stays in the hospital
14.	COM	[in Urdu] that he (doctor) is saying that, no, today you have to stay here but there is no risk. No risk
15.	COM	No risk? Euh, he is afraid
16.	DOC	If it was me [snaps fingers], they can do it now. No problem
17.	PAT	(in Pakistani Punjabi] Yes, yes, I will tell in 10–15 min
18.	COM	Eh in Urdu
19.	PAT	[in Urdu: incomprehensible]
20.	COM	Ok, he said “after ten minutes I tell—tell you”
21.	DOC	Pardon?
22.	COM	After ten minutes … he is… he is… is he (phoning?) Pakistan [incomprehensible] mobile
23.	DOC	Yes
24.	COM	After ten minutes he tell you
25.	DOC	If he decides to home … this night he will come back. Because it will be too painful. Ok?
26.	COM	Yes, yes yes yes
27.	DOC	It’s useless to return home … and if he would return home and he looks for another hospital afterwards, they will redo all the exams and they will also say that he must stay in the hospital
28.	COM	Yes
29.	DOC	So, it’s useless to refuse
30.	COM	Aaah
31.	DOC	You can discuss
32.	COM	[in Urdu] He is saying if you go home you cannot come back here. If you will go to another hospital they will examine again and you have to wait for the reports and it will take more time

#### The nature of a language barrier and the “communicative swing”

A language barrier is not an absolute concept; it can exist in a continuum of degrees or intensities (Cox 2017). The intensity of the language barrier does not only depend on the language proficiency or skills of participants to the interaction. It also depends on the communicative purpose (e.g. how specific/technical is the information sought by the doctor?); and on a set of elements which comprise contextual factors (such as stress, anxiety, and time pressure), para-verbal (e.g. how words are said) and non-verbal communicative resources (such as body posture, gestures, and the display of relevant artefacts). These elements can mitigate miscommunication resulting from language barriers, but also reinforce it.

Moreover, as the communicative purpose evolves over the course of the consultation, and the availability of communicative resources varies, a language barrier is not a static, but rather a dynamic concept which can vary in intensity over the course of an interaction. As the quality of the exchange is continuously changing in response to the changing intensity of the language barrier and the available communicative resources, and the exchange of information can be easy at one point and difficult at another point during the same interaction, a “communicative swing” can be observed (Cox 2017). For instance, while history taking typically is a language-intensive activity, palpating a patient to locate pain usually does not require many words. Moreover, in the particular medical consultation considered in this paper, the patient’s bodily position conveyed rich clinical information on the patient’s condition. In other cases (such as a potential heart failure), more linguistic resources will be required to establish a diagnosis.

Partial language barriers manifest themselves in word-finding difficulties, problems with pronunciation and/or with the understanding of utterances. In some cases, mispronounced words and ungrammatical sentences do not hamper understanding as full linguistic correctness is to some extent redundant; especially when verbal resources are complemented by non-verbal ones (e.g. as in the illustrated case, where the patient held his hand on his lower back region). In other cases, ungrammatical and mispronounced utterances do hamper understanding, especially if very specific or technical information is sought. While patients may have sufficient linguistic resources to engage in casual conversations, it is more challenging to talk about more specific terms that are not used so often in common talk.

#### False fluency

One particular risk associated with a partial language barrier is that of false fluency (Flores et al. 2012; Wadensjö 1998). While in the case of a full-language barrier misunderstandings are easily detected as the communication flow is overtly obstructed, in the case of a partial language barrier, misunderstandings are more likely to remain unnoticed. In the case of mediation by a third party (e.g. a patient companion), information may get lost in translation. Excerpt 2 illustrates how the companion sometimes seems to take up his role as an interpreter by conveying messages from the doctor to the patient and vice versa. Unfortunately, at the time of the consultation, the doctor could not observe the accuracy of the interpretation, making it very difficult to assess the quality of the communication process.

#### The medical consultation as a theatre play

Excerpt 1 illustrates how the patient’s companion initially refuses to take up the role of interpreter. In response, the doctor actively engages in role negotiation as to get across to the patient. This brings us to the issue of role distribution in a social interaction. Extensive research in this area has been carried out by Goffman (1959), who compared social interaction with theatre play, where each participant takes up a certain role. In Goffman’s view, participants to a social interaction cannot determine the role they take up in isolation from others. As such, participants to a social interaction, and by extension to a medical consultation, are part of a team of actors, by analogy with a cast of actors in a theatre setting. At the start of each interaction, individuals negotiate their roles with other participants. This implies that an individual’s role depends crucially on the context of the interaction as well as on the other participants. Throughout the consultation, participants can assume a variety of roles. Unclarities in the role distribution may give rise to confusion and miscommunication.

From this angle, the ED, where our illustrative example comes from, resembles a high-stake improvisational theatre play (Cox 2017). Individuals are interacting in an uncertain environment where they are not aware upfront of each other’s level of understanding in terms of linguistic skills as well as in terms of their reference frameworks. While the nature of the ED consultation determines that the patient has a medical complaint and that it is the doctor’s responsibility to identify the problem and propose a solution, the way to accomplish this is unplanned and unscripted. Depending on the communicative repertoire of the participants, their creativity, inspiration, and cognitive efficacy,^2^ different communication strategies and roles will be called in.

According to Goffman’s (1981) theory, a speaker can take up three different roles: the animator (the “sounding box” through which utterances are produced), the author (the individual who thought out and composed the utterances in the first place) and the principal (the individual or party whose beliefs are represented by the words uttered). A hearer, on the other hand, can be ratified or unratified (Goffman 1981). The ratified hearer is a hearer who is fully entitled to listen to the speaker; contrary to the non-ratified hearer, who is an accidental overhearer or bystander. Within a group of ratified hearers, a further distinction can be made between a “primary addressee”, the person whom the speaker focuses his visual attention on and to whom he expects to hand over the speaker role in the next turn, and the unaddressed ratified hearers, who are entitled to listen, but are not directly addressed.

Based on his behavior, some would consider the companion as an informal (non-trained) interpreter. Diverging views are discussed in the literature on which roles professional interpreters can and should take up (Hlavac 2014). The most extreme view would advocate that interpreters focus on the role of animator, in which they strictly relay the source speech in the target language, without any additions or substitutions. Patient companions providing linguistic support are however not bound by any professional code, but rather by their relationship with the person for whom they are interpreting (Hlavac 2014). Consequently, they are highly likely to take up broader roles, and to act as a principal, expressing their own thoughts and ideas.

Excerpt 2 illustrates how the patient companion switches from an animator (e.g. line 20, where he translates almost literally what the patient said before) over an author (e.g. lines 2 and 4, where he enquires about logistic details, presumably on behalf of the patient) to principal (e.g. line 15, where he explains to the doctor that the patient is afraid to clarify the latter’s behavior). The companion also takes up the role of an advocate and intercultural mediator, as he makes explicit efforts to support the negotiation process between the doctor and the patient by, on the one hand, explaining to the doctor that the patient is worried, and, on the other hand, aligning back with the doctor by telling the patient that he must stay in the hospital and that there is no risk involved.

The shift from one role to another is, however, not done in an overt or explicit way. This can generate confusion and unnoticed miscommunication, for instance if the doctor assumes that the companion is “relaying” his words to the patient in Urdu, while the companion is acting as a principal and talking about something else. An example is provided in line 32, where the companion is bringing a very different message to the patient (“If you go home now you cannot come back here”) than what the doctor initially said (implying that it is useless to go home or to look for a different hospital). The doctor, however, does not notice the inaccurate interpretation. This is also referred to as “false fluency” in the conversation (see earlier).

The doctor also switches roles throughout the conversation e.g. from “expert in charge” (line 1: “we will do the operation tomorrow”) (Lachance 2016) to “expert guide” (e.g. line 16) (Lussier and Richard 2008), when he tries to soothe the patient and convince him to undergo the surgery. Given the inability of the doctor to address the patient directly, he addresses most of the time the patient’s companion as the primary addressee; while the patient is involved in the conversation as an unaddressed ratified hearer. At other times, however, the doctor addresses the patient directly, and expects his companion to relay his question to the patient. Such switches may create confusion among the different participants to the consultation, especially in cases where the patient’s, the doctor’s and the companion’s objectives are not aligned.

The respective roles taken up by the speaker and the hearer define the “participation framework” of the social interaction, and participants rely on linguistic (e.g. code switching, pitch, volume stress and tonal quality) as well as non-verbal behavior (e.g. gestures, body orientation and touch) to try to shift participation frameworks to a more preferred one (e.g. to re-attract attention from potential hearers) (Goffman 1981). In some cases, the participation framework can be differentiated into different “stages”: Goffman (1981) described how, as in theatre, individuals’ communicative behavior may change between the “front stage” (the public sphere) and the “backstage” (the private sphere). In the ED, the “front stage” can be thought of as the consultation room where the doctors examines the patient and addresses him in a formal way. The office where the doctor fills out his files and meets other doctors and nurses can be viewed as “the backstage”. At a different level, one can discern the instances where the patient or his companion address the doctor as “front stage”; while the “space” where the patient and his companion are having informal conversations in between, in a language the doctor does not understand, can be viewed as “the backstage”.

#### Misalignment of frames

Negotiation turns particularly contentious during the treatment negotiation, when the doctor announces that the patient will need to stay in the hospital in order to get prepared for surgery the next day. The patient gets anxious and indicates he would first like to make a phone call. Subsequently, a conversation unrolls in which the doctor and the patient seem to be talking at cross-purposes: the doctor wants the patient to agree to undergo surgery, but the patient wants to call his family in Pakistan before agreeing. In the LSI literature, such instances are referred to as “misalignments in frames” or in “inferential schemata”.

These concepts derive from theories by Levinson (1992, p. 72) that posit that each “activity type” (referring to instances of communication that are goal-defined, involving people, with constraints regarding their setting, their participants, and what can be done or said within the context of that activity type) is associated with a set of inferential schemata, which participants to an activity use to interpret what is said.^3^ These inferential schemata pertain to a space of shared background knowledge and/or understanding on what the activity is about and what would be a common way to respond to what is said or done in the context of that activity. Inferential schemata are ways in which individuals organize their experiences, and they allow individuals to locate, perceive, identify, and label concrete events experiences in their daily life, mostly unconsciously. They define the principles individuals follow when deciding how to behave and communicate in particular contexts. Goffman’s (1974) term “frames” refers to a similar concept.

The complexities of communication that arise from the necessity to understand the nuances of the applicable frames or inferential schemata imply that specific knowledge and effort is required from the hearer to correctly interpret the speaker’s intention. When the hearer does not have the necessary knowledge to understand what is being implied; or if the speaker does not clearly formulate his utterance in a way that his implication can be understood, miscommunication can arise.

In the hectic and time-constrained environment of an ED for instance, where there is often little time for the negotiation of meaning, and participants to the interaction are often tired (e.g. because of long clinician shifts) and/or anxious (see earlier), effective communication requires participants to be sensitive to each other’s contextualization cues (Gumperz 1992). Where these conventions are likely to diverge because of differences in cultural or linguistic background, participants should be aware of the fragility of the communication context; and that what they say can be interpreted in a different way than it was originally intended. These differences in interpretation can trigger considerable confusion, of which the cause may be wrongly attributed or not detected at all (Gumperz et al. 1979), particularly in the case where inferential uncertainty (because of the lack of shared background on common practice and expectations) is compounded by linguistic uncertainty (because of gaps in language skills from either side).

For example, when a doctor asks a patient who visits a hospital: “What brings you here?”, he would like to hear about the patient’s complaints. A foreign patient may, however, wrongly interpret this question as the doctor wanting to know how the patient has arrived in the hospital (e.g. by which means of transport). If a doctor recommends a patient “You might want to rest for a few days”, the patient may understand this as a potential development of his condition rather than a recommendation. These instances of miscommunication are sometimes referred to as “misalignments in frames”. Frame differences between participants may surface in the conversation, but they may as well pass by unnoticed. In cases where a misalignment in frames does not alter the outcome of the consultation; it may still result in protracted consultation time.

#### “Voice of the lifeworld” versus “voice of medicine”

This particular misalignment in frames between the doctor and the patient could also be seen as an example of a conflict between the doctor’s “voice of medicine” and the patient’s “voice of the lifeworld” frame. These concepts were coined by Mishler (1984), a social psychologist. He distinguished “the voice of the lifeworld”, that is, everyday life and concerns of the patient, and “the voice of medicine”, representing the medical agenda and reasoning of the doctor. Mishler explored how these two voices interact and enter into conflict with each other during a medical consultation. He observed that the “voice of the lifeworld” frequently tries to interrupt the dominant “voice of medicine”, for example when a patient tries to bring up additional personal concerns which may or may not be related to the problem for which he is seeing the doctor. Faced with such interruptions, doctors can react in different ways: either by opening discourse to the “voice of the lifeworld”; or by keeping discourse closed, suppressing the voice of the lifeworld, and re-establishing dominance of the “voice of medicine”. Mishler felt that doctors should pay more attention to the patient’s voice of the lifeworld in the medical consultation. Even though other studies have shown that the distinction between these two worlds is not always clear cut (see for instance Atkinson 1995), Mishler’s work has brought an important theoretical contribution to the understanding of misunderstandings (Roberts 2006, p. 743).

#### Losing and saving face in the consultation

Misunderstandings can also arise from misalignments in social conventions. The incidence of a language barrier complicates the negotiation of these social conventions. Goffman (1967) invoked the concept of “face work” to describe how humans try to save their own or their counterpart’s face via politeness and/or humor, as to avoid their own or the other’s embarrassment. Attempts to save face may at times interfere with open communication.

The fact that the patient did not explain overtly (via the companion) that he was afraid, but instead insisted that he needed to make a phone call, may have been a face-saving strategy to hide his embarrassment about being anxious about a “minor operation.” Another illustration of “face-work” is presented in Excerpt 1, where the doctor took time to listen to the companion and explain to him why he needed to relay the patient’s words, rather than ordering him in an authoritative way to take up the role of interpreter. This allows the doctor to achieve alignment, rather than creating friction in the consultation. More generally, checking and displaying misunderstanding, discussing private issues and carrying out physical examinations can be considered as “face-threatening moments” (Roberts 2010, p. 21) in language discordant consultations without a common frame, potentially interfering with smooth communication.

#### Metacommunication

In response to the patient’s resistance, the doctor continues to negotiate with the patient, including by providing more technical details on the surgery. The patient is not in need of more arguments, however: he just wants to call his parents first. As the doctor fails to break the patient’s resistance before he calls his parents, the companion interrupts the conversation at some point, informing the doctor that the patient will call home.

In both cases, meta-communication is used (by the doctor and the companion respectively) to facilitate frame convergence. Van de Poel et al. (2013) highlight the importance of meta-communication in preventing or solving conflicts with patients. Meta-communication literally means communication about communication. This can happen in a verbal or a non-verbal way. Meta-communication can for instance mean that the doctor explains what he will be doing next, why, and how. It can help the patient feel more secure, at ease, and in control. Meta-communication may also be used for role negotiation. If a patient or his/her companion does not spontaneously take up the role which is preferred by the doctor, the doctor may use meta-communication (see Excerpt 1, line 6) to explain why he prefers the patient or his/her companion to take up that particular role in the interaction.

#### Noise on the phone line

The patient’s anxiety in view of a minor surgery could relate to the stress of being in an unfamiliar environment, exacerbated by the language barrier between the patient and the doctor (Meuter et al. 2015). This shows how language barriers can reinforce more general challenges to the medical interview, which do not directly relate to the language barrier itself; and that medical interviews involving language barriers are more vulnerable to different types of miscommunication than those involving native speakers only. The patient’s anxiety could as well be reinforced by his background: it is possible that due to the poor state of public health care in Pakistan, small interventions such as the removal of a kidney stone sometimes have fatal consequences and therefore have high stakes and induce anxiety. In this sense, the state of public health care in Pakistan may have added “noise on the telephone line” which the doctor was not aware of.

Goffman (1967, p. 139) introduced the metaphor of the “telephone booth bias” to describe that what patients say or do not say during a medical consultation is influenced by others or external factors. Views of others (who are not physically present) on the patient’s condition, as well as their views on good or bad behavior in society, might act on the patient’s talk during the medical encounter in a similar way as “noise on a telephone line”. Other sources of noise may be previous experiences with health care, or beliefs about alternative health care. While they may provoke miscommunication, these factors are not always visible.

## Discussion and implications for training

This paper proposes to look at clinical skills in medical interactions through the lenses of language and social interaction research to study human interaction, where the meaning of words cannot be disconnected from the social interaction they generate, and which houses them. As such, it provides several insights and methodologies that are relevant to study clinical communication from a multidisciplinary and interactional perspective.

The paper explains and illustrates, based on real-world observational data, that while many aspects of the language games that unroll during a clinician-patient interaction are not visible during the consultation, they do have an impact on its communicative and clinical outcomes. In consultations, clinicians, patients, and possible third parties present need to find a way to distribute roles, communicate questions and expectations in a context of time constraints, stress, sometimes exacerbated by the absence of a common linguistic repertoire and a lack of mutually shared background knowledge. While different types of communicative challenges become more visible in stressful medical contexts and in the presence of a language barrier (as illustrated in this paper), they are also relevant for language concordant consultations or clinical encounters in other contexts such as in pharmacies.

The proposed way of looking at medical communication can enrich clinical skills training. Looking at communication from an LSI perspective can visualize recognizable acts of human behavior which interact with and have an impact on communication processes. As such, it provides a tool to study, analyze, and discuss communication from a perspective that is complementary to the positivist clinical skills perspective, accounting simultaneously for the interactional and clinical reasoning machinery of a consultation. This allows future clinicians to better prepare for practice in a diverse environment; and experienced clinicians to reflect about their own clinical work from an angle that unveils relevant elements of the often invisible dynamics of the consultation. Such preparation and reflection is crucial, as being aware of potential pitfalls in communication is the first step to prevent or overcome them.

A potential set up for clinical skills training integrating the messages of this paper can proceed in various ways. First, students are introduced to the basic principles and a set of key theories (e.g. Wittgenstein, Goffman, Levinson, Mishler, Gumperz) in the area of LSI. Subsequently, they are encouraged to reflect on what LSI-based analysis and reflection can contribute to their clinical work. This can be done via anecdotes and more elaborate illustrations (as the ones provided in this paper). One can also proceed in a more formal way, for example by providing students with an anonymized audio-recording and the corresponding transcript (including, where applicable, the necessary translations), and requesting them to critically review the considered case both clinically and at the level of communication, by means of a written essay. Such an approach can support the development of clinical communication, clinical reasoning and analytical skills. Integrating the development of different types of skills in a training component is particularly useful at present times, where there is pressure to accelerate and shorten medical training curricula (see e.g. Custers and ten Cate 2018; Raymond et al. 2015), and where it is difficult to allot ample time and resources to courses focusing on communication alone.

In a next step, the insights gained can be applied more widely in the medical training curriculum, e.g. by developing specific case studies and role-plays that involve more challenging communicative conditions (such as patients with less communicative resources). Students that are trained to perceive communication through the lenses of language and social interaction theory, will have the repertoire to discuss such aspects—possibly based on replay of video-recorded performances in the clinical skills lab and via stimulated recall (Paskins et al. 2017). One can play around with giving students access to translations (or other elements of the interaction that are unavailable to the clinician in real-time) directly or only after they have been asked to write down a diagnosis in a clinical report or medical record, with the added feature of stimulating reflection on how much the newly available information would change the written diagnosis.

LSI-informed training is not proposed in this paper as to replace existing forms of training. Rather than a self-standing component of the training curriculum, it is a tool that can be embedded and integrated in existing training components, to highlight the interaction between, on the one hand, the positivist character of clinical reasoning and, on the other hand, the constructivist character of the actual communication process during the consultation. In this way, it complements and brings more justice to existing training methods.

## Footnotes

1 On the other hand, Silverman et al. (2011, p. 959) argue that “*Creativity lies in knowing when things are going wrong and being able to apply the skills that will enable a return to safety. The overemphasis on improvisation and creativity can damage relationships with patients*”.

2 If interactants are tired and/or nervous, their cognitive efficacy may be weaker than if they are well-rested and relaxed.

3 An example of an activity type is a consultation in the ED; another one is an informal conversation in a bar. As the participants and the setting of both activities tend to be different, the type of talk (e.g. the degree of informality) will be different as well. Patients seeing a doctor may prefer him not to tell them the same jokes as he would to his friends in a bar.
